# Abstract of the Proceedings of the Buffalo Medical Association, April 1st, 1873

**Published:** 1873-06

**Authors:** 


ART. IV.—*Abstract of the Proceedings of the Buffalo Medical Association, April* 1*st*, 1873.

Dr. Cronyn in the Chair.

Members present: Drs. Samo, Strong, Gay, Diehl, Daggett, Wetmore, Sloan, Walsh, Briggs, Bartlett and L. F. Harvey.

The minutes of the last meeting were read and approved.

The annual reports of the Secretary and Treasurer were presented to the Association, and, on motion, were accepted and placed on file.

The application of Dr. F. E. L. Brecht for membership was read.

The Association proceeded to vote on the application of Dr. A. H. Briggs for membership, presented at a previous meeting, and he was declared a member.

Dr. Sloan moved that the Treasurer call upon members whose dues extend beyond three years, and those refusing to pay in full to effect a compromise with them and cancel the balance, and report at the next meeting.

Dr. Samo : I desire to say a few words in regard to the room we are now holding our meetings in. The Society of Natural Sciences appear to have nearly monopolized it, and we are being pushed to the wall. It would seem as if we ought to have a room of our own. I do not like the way in which we are now situated. I should be in favor of taking a room somewhere else and let the present occupants have full possession, for they really need the room.

The election of officers for the ensuing year resulted in the following choice :

*President*—Dr. John Hauenstein.
*Vice President*—Dr. J. N. Brown.
*Secretary*—Dr. L. F. Harvey.
*Treasurer*—Dr. J. J. Walsh.
*Librarian*—Dr. J. B. Samo.

VALEDICTORY ADDRESS OF THE PRESIDENT, DR. CRONYN.

I could hardly be called President, as I have been absent so great a portion of the year. If I had been home I should have done but little to advance the interests of the Society, though would willingly do all in my power. Papers on subjects in practice would be of interest, but I could not give them. Perhaps a scolding would be in order, if there were more present, but people do not like to be scolded. There does not much good result from a valedictory. It is a dead letter. I think a living Society should have a permanent, comfortable room, a library, and frequent meetings, which would bring in members. We should make the meetings of interest to all.

Dr. Sloan: Why cannot a Medical Society have a library to refer to as the lawyers have, in the Law Library in this building ?

Dr. Gay : I am ashamed of the report of the Secretary—only four meetings during the year ! There are eighty physicians in the city. The fact is there is no activity in this Society. It had better expire if it cannot do more the coming year. The Society is, or should be, the thermometer of the profession. When the city had only 50,000 inhabitants thirty members were the average attendance at the meetings. The Albany Society's transactions are printed here. We will be soon wiped out, or it will be believed that we are trying to destroy each other. I will not treasure up any unpleasant occurrence that may have happened here. I am willing to put my shoulder to the wheel and try to bring the Society up again. I believe we can make it a pleasant place to come to.

Dr. STRONG: I regard a city Medical Association as indispensible to the real advancement and the true honor and dignity of the profession. Our County Society is an organization that has its uses; meeting a want by securing certain legal recognitions and relations, but its influence in promoting medical knowledge is just *nil.* We meet there but twice a year, to go through with certain legal formulas, to propose it may be, discuss and pass upon some needful enactment, perhaps to do a work of discipline. And that is about the sum of its functions. It might be fitly characterized as a Medical Police Commission. The idea of discussing medical questions, or of reporting cases, in a word, of promoting true medical culture, is quite foreign to its sessions. It is as a mere shell to its kernel, and for all purposes of information and cultivation in our Science and Art it isn't worth the snap of one's finger. He had been in it, attending faithfully upon its sessions for twenty-five years, and he could hardly recall a single thing done or suggestion made, that had much bearing on the increase of our stock of knowledge. I hesitate not to say that whatever has been done for the general advancement of medicine, by medical men in our city in an associated capacity, for the past quarter of a century, has emanated from this Association. And I point proudly to its record in this regard. Its past, at least, is secure.

Now, with such a history, shall we proclaim ourselves as degenerate sons of noble sires, by allowing this glorious Association to dwindle and die by inaction on our hands? With a population in our city of 150,000, and with from 75 to 100 medical men, as a class unsurpassed in ability and accomplishments, for its work, I should blush for my profession if such a record must be made concerning it.

For one, I must say, so benign and profitable have been these

Association meetings to me personally ; so essential do they appear to me for keeping alive the *esprit du corps* of the profession and saving it from degenerating into a mere self and pelf seeking trade, that, seems to me, apart from all motives of pride in its history, if I must forego all the social and professional advantages derivable from such an association with professional brethren, our honored calling would be largely divested of its charms for me, and I should feel tempted to make arrangements instanter for quitting it for life.

But I must yet think better of the profession of Buffalo than to anticipate any such necessity. And it strikes me that all that we need is for each one of us to cherish a sense of individual responsibility for attendance at its regular meetings, and enhancing its usefulness and honor.

Dr. Bartlett : I am much impressed with the remarks we have listened to. During the summer was often here, but could not get into the rooms. It is strange in a city of the population of Buffalo there are no reports of a Medical Society. Older physicians are derelict in duty ; they ought to give advice to the younger members. In epidemics it is very necessary that we should have reports. We have no reports in the Buffalo Medical Journal. We should have morbid specimens to exhibit. Am willing to make sacrifices to advance the interests of the Society. Not a month goes by that I do not have a case of interest.

Dr. Cronyn : Articles of interest are refused by the Journal as being too long, whilst there are very lengthy reports published from other societies.

The Association *Journal* of London is second only to the *Lancet*. The Association is composed of seventy-five members. The physicians are scientific, but not so practical as ours. I would trust ours sooner than those of England. They use all the various methods of diagnosis and insist if the patient die he must die scientifically. Every year we all promise to put the shoulder to the wheel and push the work forward. We must get young men into the Society. We must have visitors. We should have a journal to express our ideas ; let one journal have a friendly contest with the other. Have you seen the last journal? one would say ; it has an article by Gay attacking White ; another by Rochester against some one else. The London Association *Journal* fights the others continuously.

Dr. Gay : I venture to make a prediction to-night : The time will come when we shall have a large membership—honorary and corresponding. We shall have a Secretary to whom we shall pay a salary, who will maintain a correspondence all over the world, having a relation with all Societies. A journal will be conducted by the Association. The Society of Natural Sciences has obtained very much from its extended corresponding membership.

Dr. Cronyn : Without the press in these days, hardly anything can succeed. We must have a medium through which we can express our ideas. In London the meetings of the Association are held in Society Hall. Sometimes one hundred are present ; frequently twenty or thirty of these will not be practictioners ; the discussions are so interesting, others are attracted to the meetings.

Dr. Gay : I think the editor of the Journal never has refused to publish the proceedings.

Dr. Bartlett : Our proceedings are frequently noticed in foreign journals.

Dr. Gay : I saw quotations of Dr. Bartlett's paper on Cerebro-Spinal-Meningitis in a foreign journal.

Dr. Strong : Years ago our discussions were highly spoken of in all journals.

Dr. Bartlett : I consider an Association journal very necessary. Look at the old numbers of the Buffalo Medical Journal, and you will find of how great value our proceedings were. Now we absorb all and give nothing.

Dr. Strong : The first thing subscribers to journals look for are the Society discussions. They are the most interesting to country practitioners.

Dr. Cronyn : It must be always so ; discussions between the old and the young, on the true and the false, are instructive. Conclusions of Societies are more valuable than papers.

Dr. Strong : We have it in our power to make a united Society and have large meetings. We must have a journal of our own, if the editor of the Buffalo Medical and Surgical Journal declines to publish our proceedings. We must be here and by reports of andcases in discussions make the meetings interesting.

Dr. GAY: Much is said in meetings that would not look well in print. Do not think the editor will refuse what is right. An editor must use discretion.

On motion, adjourned.

